# Whole-Genome Sequencing for Risk Assessment of Long-term Shiga Toxin–producing *Escherichia coli*

**DOI:** 10.3201/eid2004.131782

**Published:** 2014-04

**Authors:** Johannes K.-M. Knobloch, Stefan Niemann, Thomas A. Kohl, Ulrich Lindner, Martin Nitschke, Friedhelm Sayk, Werner Solbach

**Affiliations:** University Hospital Schleswig-Holstein, Lübeck, Germany (J.K.-M. Knobloch, U. Lindner, M. Nitschke, F. Sayk, W. Solbach);; Research Center Borstel, Borstel, Germany (S. Niemann, T.A. Kohl);; German Centre for Infection Research (DZIF), Partner Site Hamburg–Lübeck–Borstel, Germany (J.K.-M. Knobloch, S. Niemann, U. Lindner, W. Solbach)

**Keywords:** risk assessment, whole-genome sequencing, treatment, Shiga toxin–producing Escherichia coli, STEC, long term carrier, bacteria

**To the Editor:** Long-term carriage of Shiga toxin–producing *Escherichia coli* (STEC) can greatly affect the social and work lives of infected patients. We describe the use of whole-genome sequencing to assess the risk from long-term STEC carriage in a patient who had been denied surgery because of the infection.

On August 18, 2013, a 64-year-old woman reporting to be a carrier of STEC since March 2013 contacted the University Medical Center Lübeck, Lubeck, Germany, seeking decolonization therapy that had been provided to long-term STEC carriers during the 2011 STEC O104:H4 outbreak (*1*). STEC had initially been identified in the patient during an episode of watery diarrhea. She currently had gonarthrosis grade III, indicating the need for a total knee endoprosthesis; however, the responsible orthopedic department had denied surgery because of the potential risk for development of STEC-associated hemolytic uremic syndrome (HUS) caused by the perioperative use of antimicrobial drug prophylaxis. The patient was also rejected for surgery at another orthopedic clinic. Because of this STEC-associated restriction, the patient requested decolonization therapy.

Before responding to the request, we asked the patient to provide a fecal sample for STEC strain typing. A sample provided on August 22, 2013, was confirmed positive for STEC by culturing an STEC strain on MacConkey agar (bioMérieux, Marcy l'Etoile, France) that did not grow on selective agar (CHROMagar STEC, Mast Diagnostika, Reinfeld, Germany) optimized for the detection of classical enterohemorrhagic *E. coli* strains. Total DNA was extracted from the isolate, and a sequencing library was generated by using the Nextera XT Sample Preparation Kit (Illumina, San Diego, CA, USA). Sequencing was performed (MiSeq Benchtop Sequencer, Illumina) in 2 batches of paired 250-bp sequencing runs. Sequencing reads were further analyzed by using the CLC Genomics Workbench software package (CLC bio, Aarhus, Denmark). De novo assembly resulted in 120 contigs with an average length of 44,331 bp (N50 = 126,317 bp). A predefined dataset of 2,456 sequences was aligned with the generated contigs in a single step by using BLAST (http://blast.ncbi.nlm.nih.gov/Blast.cgi) to determine the alleles and subtypes of genes usually used for *E. coli* and STEC strain typing and for seropathotype detection.

Presence of a Shiga toxin subtype 1a with >99.9% and 100% identity to the *stx*1aA and *stx*1aB subunit genes, respectively (GenBank accession no M19473.1), was confirmed. The STEC strain carried genes with high homology to the O91 antigen–encoding operon (GenBank accession no. AY035396.1) and the H14-flagellin gene (GenBank accession no. AY249998.1). This observation was confirmed by a 100% sequence identity of a 643-bp fragment of the *gnd* gene of the sequenced strain with that of a *gnd* reference sequence of STEC O91:H14 (www.corefacility.ca/ecoli_typer). These 2 sequences are different from the *gnd* sequence of reference strain STEC O91:H21. In vitro multilocus sequence typing (*2*) identified sequence type (ST) 33. These data were used for risk assessment.

Only strains displaying serotype O91:H21 and a single O91:H10 isolate have been associated with HUS in humans (*3*,*4*). ST33, identified in the patient in this study, has not been associated with HUS in humans despite being the most frequently identified ST of O91 STEC strains in humans (*3*). In addition, the identified strain carried only Shiga toxin 1a, whereas the HUS-associated strain HUSEC034 of serotype O91:H21 carried Shiga toxins 1a, 2a, and 2d (*5*). This data indicated the patient strain was a seropathotype D strain (*6*) with a relative low risk for HUS development in the patient.

The assumption that the patient strain had low pathogenicity was further corroborated by the analysis of additional marker genes (*6*–*9*) indicating the lack of pathogenicity islands associated with high virulence of STEC in humans. None of the 25 marker genes suggested for the LEE locus or pathogenicity islands OI-36, OI-43, OI-44, OI-48, OI-50, OI-57, OI-71 or OI-122 were identified in the patient strain, whereas most of these markers could be detected in highly pathogenic STEC/enterohemorrhagic *E. coli* strains used to establish the method for identifying markers (Technical Appendix Table).

After completing the STEC risk assessment, we advised the patient’s general practitioner that antimicrobial drug prophylaxis could be administered for surgery with a low calculated risk for HUS development, as observed for other non-O157 strains (*1*,*10*). In addition, we described our experience with 4 long-term carriers of STEC O91:H14 strains; the patients had been decolonized of STEC by the use of azithromycin decolonization therapy (data not shown).

The patient was added to a waiting list for surgery, and she elected to receive azithromycin as experimental decolonization therapy while awaiting surgery. Azithromycin was administered orally for 3 days (500 mg/day); fecal specimens on post-treatment days 7, 14, and 21 were negative by Shiga toxin ELISA. In addition, an *stx*-specific PCR using enrichment broth confirmed the sustainable eradication of the STEC infection. Our findings show that whole-genome sequencing can be used in the diagnostic process for long-term STEC carriers and might extend or replace other methods used for risk assessment (*6*–*8*,*10*) and treatment decision guidance.

Technical Appendix Marker genes identified in clinical strains and the patient strain during whole-genome sequencing assessment of the risk for development of Shiga toxin–producing *Escherichia coli* (STEC)–associated hemolytic uremic syndrome in long-term Shiga toxin–producing *Escherichia coli* infection.
