# Supplementary material for: Whole-Genome Sequencing for Risk Assessment of Long-term Shiga Toxin–producing Escherichia coli
Source: Emerg Infect Dis. 2014 Apr;20(4):732–3. doi: 10.3201/eid2004.131782 (PMC3966390; doi:10.3201/eid2004.131782)
Supplement: Technical Appendix — Marker genes identified in clinical strains and the patient strain during whole-genome sequencing assessment of the risk for development of Shiga toxin–producing Escherichia coli (STEC)–associated hemolytic uremic syndrome in long-term Shiga toxin–producing Escherichia coli infection. [file 13-1782-Techapp-s1.pdf]

Article DOI: http://dx.doi.org/10.3201/eid2004.131782

Whole-Genome Sequencing for Risk Assessment of Long-term Shiga Toxin–producing *Escherichia coli*

Technical Appendix

Technical Appendix Table. Presence of marker genes in 2 independent clinical strains and the patient strain in a whole-genome sequencing assessment of the risk of long-term Shiga toxin–producing *Escherichia coli* infection*

Strain	eae (Z5110)	ent/expL2 (Z4326)	nleB (Z4328)	nleE (Z4329)	nleG2–3 (Z2149)	nleG6 (Z2150	nleG5–2 (Z2151)	nleB2 (Z0985)	nleC (Z0986)	nleH1–1 (Z0989)	nleD (Z0990)	nleG (Z6010)	nleF (Z6020)	nleH1–2 (Z6021)	nleA (Z6024)	nleG2–1 (Z6025)	nleG9 (Z2560)	Z2098	Z2099	Z2121	ureD (Z1142)	espV (Z1387)	espK (Z1829)	espN (Z1824)	espM1 (Z2565)
O26:H11	+	+	+	+	+	+	+	–	+	+	–	+	+	+	+	+	+	+	+	+	+	+	+	+	+
O146:H21	–	–	–	–	–	–	–	–	–	–	–	–	–	–	–	–	–	–	+	–	+	–	–	–	–
O91:H14	–	–	–	–	–	–	–	–	–	–	–	–	–	–	–	–	–	–	–	–	–	–	–	–	–

*Marker designation and locus tags in the annotated genome of enterohemorrhagic *E. coli* O157:H7 strain EDL933 (GenBank accession no. AE005174.1) are shown. Gray shading indicates the patient strain. +, detection of the respective sequences in the contigs of de novo assembled whole-genome sequencing procedures.
